# Co‐Development and Validation of a Patient‐Reported Experience Measure for Trans and Gender Diverse Young People: A Multi‐Stage Participatory Study Protocol

**DOI:** 10.1111/hex.70678

**Published:** 2026-05-03

**Authors:** Sasha Bailey, Isabel Zbukvic, Alex Dalton, Steph Odoi, Emily Clarke, K. A. McKercher, Ken Pang, Alessandra Chinsen, Maria Pallotta‐Chiarolli, Alan Bailey, Penelope Strauss, Kate Filia, Caroline Gao, Skye Barbic, Sarah Bendall, Sian Lloyd, Andrew Chanen, Nicholas Fava, Eóin Killackey, Susanne Prosser, Vikki Ryall, Lou Kerley, Corrinne T. Sullivan, Magenta Simmons

**Affiliations:** ^1^ Centre for Youth Mental Health The University of Melbourne Parkville Victoria Australia; ^2^ Orygen, The National Centre of Excellence in Youth Mental Health Parkville Victoria Australia; ^3^ Beyond Sticky Notes Wangal Country Sydney Australia; ^4^ Clinical Sciences, Murdoch Children's Research Institute Australia; ^5^ Department of Paediatrics University of Melbourne Australia; ^6^ Arts and Education Deakin University Burwood Victoria Australia; ^7^ Australian GLBTIQ Multicultural Council St Kilda Australia; ^8^ The Kids Research Institute, Perth Australia; ^9^ School of Population and Global Health University of Western Australia Nedlands Australia; ^10^ Faculty of Medicine University of British Columbia Vancouver British Columbia Canada; ^11^ Foundry, Providence Health Care Vancouver British Columbia Canada; ^12^ School of Public and Preventive Medicine Monash University Australia; ^13^ Transcend Brunswick Victoria Australia; ^14^ headspace National Youth Mental Health Foundation Australia; ^15^ School of Social Sciences Western Sydney University Penrith New South Wales Australia

**Keywords:** adole scents, healthcare experience, healthcare quality, participatory methods, patient‐reported experience measure, Trans and gender diverse

## Abstract

**Background:**

Trans and gender diverse (trans) young people experience higher rates of physical and mental ill‐health due to chronic exposure to gender minority stress. Consequently, trans young people report higher health and mental healthcare service utilisation. Disconcertingly, negative experiences of healthcare services are prevalent among trans young people, especially those with additional marginalised identities and backgrounds who experience multiple forms of marginalisation. Patient‐reported experience measures (PREM) are auseful tool for improving patients' healthcare experiences however existing PREMs are not relevant, appropriate, nor inclusive of the unique healthcare experiences of trans young people, especially those facing additional forms of marginalisation.

**Objective:**

This study will co‐develop and validate a PREM for trans young people aged 12 to 25 years attending health and mental healthcare services. This PREM will capture how healthcare experiences should affirm all aspects of trans young people's identities in health and mental health services, including but not limited to their gender.

**Design:**

Underpinned by a ‘co‐production’ framework, the proposed study comprises four stages informed by a Lived Experience Advisory Group (LEAG) made up of eight trans young people from across Australia. Stage 1 is a scoping review of qualitative studies exploring the experiences of marginalised young people using healthcare services. Stage 2 is semi‐structured, one‐to‐one interviews with multiply marginalised trans young people aged 12 to 25 years in Australia (*n* = 30) and healthcare professionals of trans young people in Australia (*n* = 30). Candidate PREM items generated from Stages 1–2 will be appraised in a multi‐stakeholder modified e‐Delphi Consensus Survey (*N* = 90; Stage 3) comprising multiply marginalised trans young people (*n* = 30), healthcare professionals (*n* = 30), and parents/caregivers of trans young people (*n* = 30). Lastly, in Stage 4, PREM items will be reviewed by trans young people in two cognitive debriefing focus groups (*N* = 14) to improve clarity, understandability, and interpretation.

**Discussion:**

This study will co‐produce and validate a PREM to effectively measure the quality of trans young peoples' experiences utilising health and mental healthcare services. The PREM will subsequently be implemented into integrated youth health services as part of a multi‐staged quality improvement project evaluating an integrated gender service model of care operating in Victoria, Australia.

**Patient or Public Contribution:**

The Whole of Self Affirming Care Lived Experience Advisory Group together with trans members of the research team in designated peer and non‐peer research roles have contributed to the design of the present study protocol and corresponding manuscript. These individuals will also contribute to analysis, interpretation, and write‐up of all subsequent data and outputs.

## 1 Introduction

Trans and gender diverse young people (henceforth, respectfully, ‘trans’) report higher prevalence rates of physical [1, 2, 3, 4] and mental [5, 6] ill‐health service use than their cisgender peers. These health inequities stem in part from chronic and daily exposure to gender minority stress [7, 8], that is, those forms of stigma and prejudice directed toward individuals who are not cisgender, that is, do not have a gender congruent with their sex presumed at birth [9]. Arising from cisnormativity, gender minority stress can manifest internally (proximally) within trans young people, including negative self‐regard and internalised self‐stigma, and externally (distally), for example, interpersonal discrimination, rejection, and violence [10].

Trans young people from multiply marginalised identities, experiences, backgrounds, and values experience multiple forms of stigma and discrimination in addition to that stemming from norms of cisnormativity [11]. This can be explained by Kimberlé Crenshaw's notion of intersectionality [12], which posits that individuals from multiply marginalised social positions encounter intersecting, interlocking structures of oppression that produce unique forms of marginalisation. Where Crenshaw's original treatise was developed to explain the interlocking systems of racism and sexism that produce the oppression of Black women [12], scholars have since developed intersectionality theories for trans health research which consider multiple intersecting structures of domination such as heteropatriarchy, white supremacy, and colonialism, in addition to cisnormativity [13]. It is important to note that intersectionality asserts a constitutive model of oppression rather than an additive model of oppression; the explicit naming of intersecting power relations is critical for intersectionality theory [13].

Unsurprisingly, in response to these health inequities, trans young people also report higher utilisation of health and mental healthcare (‘healthcare’) services than their cisgender peers [14, 15, 16]. However, many trans young people encounter negative experiences when receiving and utilising healthcare services [17, 18, 19]. For example, healthcare professionals who ‘deadname’, misgender, and mock their preferred language used to describe themselves [20, 21]. Furthermore, many healthcare professionals do not understand experiences of gender diversity [19] and are not competent in providing gender care [22, 23], which can leave trans young people without the information and resources they need to meet their healthcare needs [24]. Additionally, there is increasing awareness that many trans people report that healthcare services are either trans affirming but not affirming of the other parts of their identity, or affirming or other parts of their identity but not affirming of them being trans. Consequently, multiply marginalised trans young people report having to compromise part of their identity when accessing healthcare. For example, a small qualitative study of six trans people of colour observed many participants talking about the ways in which their racial, ethnic, and cultural backgrounds sparked apprehensiveness from healthcare professionals, including presumptions of one's gender affirmation to date and a lack of visibility regarding outcomes of gender affirming surgeries for trans people of colour specifically [25]. Systematic approaches to improving healthcare experiences among trans young people, particularly those navigating multiple intersecting structures of oppression, is a critical research gap.

Improving patient safety remains a global health priority for health sector policies and programs, as identified by the World Health Organisation. Within the Australian healthcare system particularly, patient safety, experience, and effectiveness are core elements of quality of care [26]. For this reason, many healthcare services in Australia mandate the use of ‘patient‐reported experience measures' (PREM). These PREMs are validated measures that reflect patients, families, and healthcare providers core components of care, and are used to improve healthcare service planning, delivery, and evaluation [27, 28, 29]. A seminal review conducted by Ambresin and colleagues (2013) has previously identified several core elements of youth‐friend healthcare service provision have been identified previously in, including staff attitude, communication, and competency [30]. Moreover, a range of PREMs are available for use with general populations of young people. However, a core criticism across these available tools is how they have not given appropriate weight to the voices of young people themselves during the development process [31]. Moreover, universal PREMs administered to all young people may underrepresent the voices of young people from marginalised identities, experiences, and identities [32], such as trans young people, thereby reducing cultural responsiveness and accessibility. A patient‐reported outcome measure (PROM), the Gender‐Q Youth [33] is readily available for use; however, there are no existing PREMs, that have been developed specifically to reflect the healthcare experiences of trans young people, their families, and healthcare providers. Importantly, no measures have used a co‐production model in development.

This paper outlines the prospective protocol of a participatory research study that aims to co‐create and validate a PREM for evaluating the extent to which healthcare experiences affirm all aspects of identities, experiences, backgrounds, and values of trans young people aged 12 to 25 years, including but not limited to their gender (‘whole of self‐affirming care’; henceforth, ‘WOSAC’).

## 2 Methods

### 2.1 Overview

The proposed project follows an adapted multi‐staged approach to co‐developing measures for young people, previously used to develop a youth‐centred measure capturing ‘function’ in young people accessing mental health and substance use healthcare services [34]. An intersectional approach to trans health as outlined by Wesp et al., (2019) is embedded throughout [13]. Firstly, this project will conduct a scoping review of qualitative research regarding marginalised young peoples' experiences receiving and utilising healthcare services, and qualitative interviews with multiply marginalised trans young people and healthcare professionals of trans young people, to generate candidate PREM items. Item generation will be strengthened with iterative and continuous LEAG input and piloting throughout. Candidate PREM items will be piloted and validated through a multi‐stakeholder modified e‐Delphi consensus survey with multiply marginalised trans young people, parents/caregivers of trans young people, and healthcare professionals involved in the physical and/or mental healthcare of trans young people. Final PREM items will be reviewed for clarity and understandability via cognitive debriefing focus groups with multiply marginalised trans young people.

**Figure 1:**
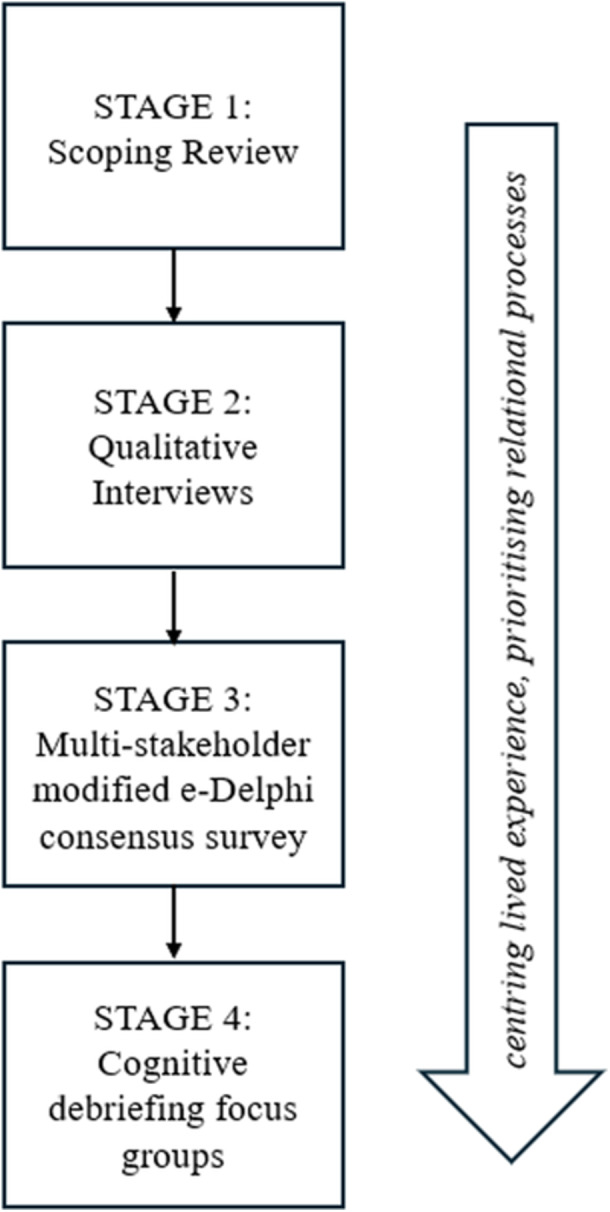
Overarching structure of the proposed project to co‐develop and validate a patient‐reported experience measure for trans young people.

Figure 1 provides a visual summary of the four stages comprising the proposed research study.

## 3 Governance

The present project was conceptualised in accordance with McKercher (2020)'s trauma‐informed guidance for undertaking participatory research which moves from consultation or designing for young people towards ‘co‐design’ and ‘co‐production’ with them [35]. Aligning with this framework, from project inception, the project team has sought to share power, build capabilities, prioritise relationships, and use participatory means with trans young people across various cyclical stages of co‐planning, co‐discovering, co‐designing, co‐delivering, and co‐evaluating. To support our co‐production approach, This project is governed by three governance and advisory bodies, each of which actively involve trans young people: a Core Working Group, Lived Experience Advisory Group, and Project Steering Group.

The Core Working Group (CWG) is responsible for the day‐to‐day management of the project and consists of one trans research fellow and project manager, two part‐time trans youth peer researchers, and two full‐time cis senior researchers. Peer researchers are employed based on their desire and ability to draw on their lived experience to undertake research rather than professional qualifications. For peer researchers who do not have formal qualifications or training, all relevant training is provided either internally by project staff or externally through training courses. Peer researchers in this study are provided with dedicated lived experience training, which includes ongoing professional development regarding LE as a discipline, opportunities to run groups, and peer wellbeing debriefs. The CWG is a ‘small circle’ [35] core co‐design team that meets weekly to discuss research activities related to the project. CWG peer researchers are trans youth in paid employee positions providing professional research assistance in addition to sharing their lived experience to inform the project.

CWG meetings are convened using a ‘transformational convening’ approach rather than ‘transactional convening’ approach [35]. This approach is characterised by active listening, proactive power sharing, and cultivating multiple ways for information to be shared and processed. To challenge power differentials [35], there is no set convenor for CWG meetings; CWG members collectively promote safety and reflexivity and work together to prioritise relational processes. CWG meetings aim to foster connection between CWG members and encourage safe, flexible ways of approaching project tasks and needs, rather than merely focus on getting through a list of pre‐determined tasks [35]. Meetings facilitate ad‐hoc check‐ins, actively promote power‐sharing, and champion consent‐based trust‐building processes. All CWG members actively promote a trans‐affirming workplace and challenge unequal power dynamics rather than accept them [35]. All meetings commence with an update from/regarding the LEAG as a way of meaningfully centring embodied trans knowledges [13].

Utilising various modes of amplifying underserved voices and representation [13] (e.g., purposively inviting expressions of interest, also colloquially referred to as ‘shoulder taps’), CWG peer researchers have recruited and established a Lived Experience Advisory Group (LEAG) comprising 8 to 10 trans young people aged 14 to 30 years. Subsequent LEAG recruitment rounds to fill vacancies were informed by consultation with current LEAG members about which voices they thought were missing from the group and project overall. One CWG peer researcher holds a ‘Lived Experience Involvement Coordinator’ role. Both CWG peer researchers are responsible for liaising with LEAG members out of session, creating a continuous communication loop before and after meetings. Both CWG peer researchers are involved in running meetings, with the Lived Experience Involvement Coordinator primarily chairing and facilitating LEAG meetings with the second CWG peer researcher assisting with minute‐taking and Zoom chat function moderation. At the start of LEAG meetings, members are invited to use a colour‐coded traffic light system (i.e., red, yellow, green) to indicate they are feeling about the meeting ahead, allowing co‐facilitators to independently check‐in appropriately. Throughout meetings, co‐facilitators remind LEAG members to use the ‘private message’ function of Zoom if they need additional support. Outside of meetings, LEAG members have the option to have confidential one‐on‐one chats with the Lived Experience Involvement Coordinator regarding their involvement with LEAG. The LEAG meet monthly for the duration of the project to directly inform decision‐making. LEAG members are reimbursed for preparation and meeting time.

A Project Steering Group (PSG) has also been established, comprising CWG members and both Chief Investigators and Associate Investigators, including experts by profession (e.g., health care professionals, senior researchers, policy advisors, service designers, leaders of community organisations), including trans investigators. PSG meetings were held monthly during the planning and early phases of the project, before moving to bimonthly meetings. LEAG meetings are deliberately scheduled before Project Steering Group meetings, and two LEAG members attend, to ensure decisions are centred on lived experience input. When no LEAG members are available to attend the PSG meeting or during months when the PSG does not meet, two members are invited to attend a CWG meeting to ensure their link to the study is maintained. A consensus decision‐making resource developed by Seeds for Change will be used in the event that disagreements or conflict advice arise (e.g., differences of opinion between LEAG and PSG members). Most PSG members will be in salaried positions and contribute their time in kind within work hours. Staff employed on the project (peer researchers and project manager) will be paid salaries. LEAG members will comprise reimbursed volunteers and will be reimbursed for both preparation and meetings times. Irrespective of the way in which members are compensated financially, we will aim to provide mutually beneficial opportunities, with a strong emphasis on career development for employed staff and tailored opportunities for all members. This study has received ethics approval from the University of Melbourne Human Research Ethics Committee (reference number: 2024‐29368‐61226‐7).

Table 1 below provides an overview of the governance bodies and their respective roles and responsibilities.

**Table 1:** Overview of the lived experience‐centred governance bodies undertaking the proposed project.^a^

Governance body	Membership	Roles and responsibilities	Meeting frequency
Core Working Group (CWG)	Trans researchers in peer and non‐peer roles Youth mental health researchers with expertise in implementation science, health services research, and participatory research methods	Lead day‐to‐day project and research operations	Weekly
Lived Experience Advisory Group (LEAG)	Eight to 10 trans young people aged 14–30 years living in Australia with marginalised identities, experiences, backgrounds, in addition to encountering cisnormativity	Share lived experiences‐driven guidance to enable project decision‐making to best reflect the experiences, needs, desires, and concerns of trans young people	Monthly plus ad‐hoc (out‐of‐session) as required
Project Steering Group (PSG)	Youth mental health professionals, including Allied health and medical professionals Paediatrician specialising in gender affirming care Representative from National Australian community organisation serving parents and carers of trans young people Representative from state/territory‐based (Victoria) Australian community organisation serving trans people living in Victoria Representative from national specialist co‐design, co‐production and service design professional Representative from Australian community organisation advocating for the health and rights of multicultural and multifaith gender and sexuality diverse people Representative from state/territory‐based (Victoria) community organisation serving multicultural and multifaith young people	Share practice wisdom and experiential evidence from relevant community sector to ensure project decision‐making aligns with agendas being pursued in relevant sectors/industries	Bi‐monthly

^a^ Characteristics and meeting frequencies of the governance bodies outlined in Table 1 are subject to change depending on the project's needs and requirements over time.

## 4 Positionality Statement

Our multidisciplinary research team includes neurodiverse individuals from a range of racial, ethnic, cultural, and professional backgrounds, including identities that have been historically and are currently marginalised. Approximately one‐third of team members identify as trans and gender diverse, including binary and non‐binary gender identities. These intersecting lived and professional experiences shape how we understand structural inequity, identity, and access to care, particularly within health and mental health contexts. Team members aspire to allyship with one another, acknowledging that positions of power and oppression are dynamic and relational. We acknowledge the multitude of ways in which people may experience and actualise their transness and we strive to resist trans normativity, including over medicalisation in health and mental health contexts.

We share a commitment to upholding human rights through personalised, socially inclusive, and meaningful care, which informed our choice of topic, design, and planned methodological approaches. We approach knowledge production from an intersectional perspective, recognising that experiences are shaped by overlapping systems of power and oppression. This in turn orients us toward centring equity and justice, including epistemic justice, in our analyses.

## 5 Study Design

### 5.1 Stage 1: Scoping Review

A systematic scoping review [36] will be undertaken of qualitative studies exploring the experiences of marginalised adolescents receiving and utilising health and mental healthcare services. Comprehensive detail regarding the protocol for this scoping review has been prospectively published as a pre‐print in an open‐access platform [37] and is currently being reviewed for publication at a peer‐reviewed academic journal. The decision to focus exclusively on qualitative studies was made to ensure that identified factors reflected the real‐world experiences of trans young people themselves, not their parents/caregivers and/or healthcare professionals, as is commonly the case with research examining the quality of healthcare receiving by younger individuals [27, 28, 29].

CINAHL, MEDLINE, Embase, Emcare, and PsycInfo will be searched for eligible articles (2005‐2025). synthesise what is known from The SPIDER approach [38] will generate search logic: ‘*Sample’* (multiply marginalised youth aged 12‐25 yrs), ‘*Phenomenon of Interest’* (receipt/utilisation of primary and mental healthcare services) ‘*Design’* (qualitative studies), ‘*Evaluation’* (young people's first‐hand accounts), ‘*Research Type*’ (original peer‐reviewed qualitative research) [39].

Title/abstract screening will be conducted in ASReview [40], a machine learning assisted screening tool. A single reviewer will screen records until either i) 50% of records screened, or ii) a data‐driven ‘stopping rule’ wherein title/abstract screening was automatically ‘stopped’ when 100 consecutive irrelevant records were consecutively screened. Stopping rules were determined based on size of dataset, visual inspection of data set, and past experiences with complex phenomena reviews, as is convention [39]. Hence, a conservative, yet arbitrary, threshold of 50% screened coupled with the data‐driven approach provides a robust rule to minimise relevant study loss while leveraging screening efficiency. A second reviewer will manually screen a random 10% of records (i) screened by first reviewer and (ii) records not seen by the first reviewer. Using Covidence, one reviewer decision will review 100% of full‐text records and perform data extraction on included studies, with a second reviewer screening a random 20% equivalent of all full‐texts and extracted articles, respectively.

In brief, this exploratory scoping review is designed to identify key factors which impact adolescents' experiences of receiving healthcare (e.g., positive experiences, negative experiences). These factors will be identified and ‘extracted’ using NVivo qualitative analysis software. Summative content analysis will be conducted in NVivo to Using the query‐ and matrix‐ functions of NVivo, Summative content analysis will be conducted in NVivo to synthesise findings.

## 6 Stage 2: Qualitative Interviews

One‐hour semi‐structured interviews with multiply marginalised trans young people and healthcare professionals with experience working with this population will be undertaken. Interviews will explore trans young people's and healthcare professionals' accounts and conceptualisations of WOSAC and elicit recommendations about how services can be more inclusive and responsive to multiply marginalised young people in the spirit of WOSAC. Interview guides will be designed in collaboration with LEAG members. The CWG peer researchers will conduct all interviews, where possible, or else by another CWG member. Senior researchers from the CWG and PSG with expertise in conducting qualitative research will upskill and train peer researchers to conduct interviews. This will include workshop sessions planning interviews, roleplaying interview scenarios, and offering the opportunity to co‐reflect on the process of conducting the interviews after they take place.

Up to 30 interviews will be conducted with multiply marginalised trans young people living in Australia. To be eligible to participate in an interview, interviewees will be aged 12 to 25 years, living in Australia, and self‐identify as having experienced disadvantages because of ‘systems’ that exclude or create barriers for people whose experiences are different to the ‘mainstream’ majority. For example, prospective participants may be trans young people of colour, neurodivergent trans young people, and trans young people living with disability. Young trans interviewees will be recruited through ‘trusted sources’ via community partners, including Transcend Australia, Centre for Multicultural Youth, and Australian GLBTIQ Multicultural Council.

Up to 30 interviews will also be conducted with Australia‐based healthcare professionals in paid roles with experience working with multiply marginalised trans young people. These include traditional healthcare professional roles such as psychologists, psychiatrists, and general practitioners, as well as emerging healthcare professional roles such as peer workers and peer navigators.

In line with neuro‐affirming and inclusive research principles, interview questions will be provided to participants in advance. This approach reduces processing demands, supports diverse communication styles, and mitigates the privileging of rapid verbal recall inherent in traditional interview formats. We hope that by providing questions beforehand, we will enhance participant comfort, autonomy, and the depth of responses, thereby improving both equity and data quality. These questions will be designed by a peer researcher and senior CWG researcher. In brief, youth interviews will explore general experiences of identity affirmation in‐person and offline, as well as those specific to health and mental health contexts. Healthcare professional interviews will explore past experiences of and perspectives about addressing factors or ingredients of care that affirm all aspects of a young person, including at individual‐, service‐, built environment‐, online environment‐, and professional/governing body‐levels. Using Zoom video conferencing software [41], all interviews will be conducted online and audio recorded. Zoom‐generated recordings will be professionally transcribed. Participants will be invited to opt‐in to review their final de‐identified transcription (‘member‐checking’) within 2 weeks of the interview. Finalised transcripts will be verified by at least one trans member of the research team and subsequently imported into NVivo Qualitative Analysis software for formal analysis [42].

To centre embodied trans knowledge [13] and strengthen prospective insights with trans lived experiences and perspectives [35], at least one trans member of the research team will analyse each transcript using a reflexive thematic analysis approach [43]. Coding will be conducted by a trans member of the research team using an iterative, reflexive approach across transcripts from both cohorts. Codes will be developed inductively through close alignment with the data, rather than through a predefined codebook. Ongoing discussions with other team members will support crticial reflection and analytic depth, with the aim of enriching interpretation rather than achieving coding consensus. NVivo will be used to organise the data, but analysis will remain a researcher‐driven, interpretive process.

## 7 Stage 3: Modified e‐Delphi Consensus Survey

The CWG will generate candidate PREM items in iterative collaboration with the LEAG. Informal summary‐style reports of key factors affecting the quality of (trans) young people's healthcare experiences identified in Stage 1 (scoping review) and Stage 2 (interviews) will form the basis of this discussion. CWG workshopping sessions will be iteratively summarised and presented to the LEAG for ongoing input and item refinement.

Once a formal series of candidate PREM items are curated, during a LEAG monthly meeting, LEAG members will be asked to identify which candidate PREM items fall within aims, scope, and rationale of the WOSAC PREM measure. During this process, LEAG members will be invited to endorse items, reword unclear items, delete irrelevant or repetitive items, or add items for enhanced comprehensiveness.

A multi‐stakeholder modified e‐Delphi consensus survey [44, 45] will be used to appraise the overall content validity of the resulting WOSAC PREM items, as perceived by trans young people, their parents/carers, and their healthcare providers. Specifically, Delphi panellists will comprise a maximum total of 90 participants, including up to 30 multiply marginalised trans young people aged 14 to 25 years, 30 healthcare professionals with experience working with trans young people with intersecting disadvantage, and 30 family members, parents, or carers of trans young people. This target panel size of 90 participants accounts for 50% attrition rate or data missingness, resulting in 45 participants' total, within the optimum range of final rounds of a consensus‐rich Delphi study [46]. Due to data being collected remotely rather than in‐person (i.e., the Delphi is an online survey), consent for trans young people aged 12 or 13 years old is not possible (unlike Stage 2 interviews which have the option to be run in‐person).

With reflexive guidance on sampling provided by LEAG and PSG members, broad intersectional diversity among Delphi panellists will be sought including but through purposive sampling. This diversity is not limited to trans young people of colour, trans young people living with disability, neurodivergent trans young people; allied health professionals psychology, psychiatry, general practice, peer navigation; and chosen family, parents, and carers of trans young people.

Delphi panellists will be invited to express interest via the trusted community partner organisations described above along with advertising on Orygen's social media account. Following CWG and LEAG consultation, invitations will be issued via email to eligible panellists, with reminder emails sent to unresponsive candidates. Confirmed Delphi participants will be presented a plain language summary of the purpose of the study, the definition of the proposed WOSAC PREM, and background information about the study development to date. An Excel spreadsheet of interested Delphi panellists will be generated, including indication of non‐responsiveness or attrition. LEAG, CWG, and PSG members will not be eligible to participate in this Delphi study.

Up to three Delphi rounds will be held to achieve optimal consensus. Each Delphi round will present participants with all candidate PREM items distilled in preceding stages. Response options will be ‘Very Relevant’, ‘Relevant’, ‘Neutral’, ‘Irrelevant’, and ‘Very Irrelevant’. Responses will be dichotomised such that ‘Very Relevant’ or ‘Relevant’ will be coded as ‘Yes Vote’ and ‘Very Irrelevant’, ‘Irrelevant’, and ‘Neutral’ responses will be coded as ‘No Vote’. An a priori consensus threshold will be defined as greater than or equal to 80% of the entire multi‐stakeholder cohort casting a ‘Yes Vote’. Items attracting less than 80% ‘Yes vote’ at any panel will be excluded. Five‐point Likert scales are the most commonly used scale response options in Delphi studies [44]. Though there is little agreement on the use of neutral or midway options in Delphi consensus measurement [44, 47], our treatment of neutral as part of a No vote is consistent with a previous Delphi study developing suicide prevention guidelines for gender and sexuality diverse young people [48]. This percentage level consensus is defined in accordance with the median percentage level consensus observed in a scoping review of the consensus‐reaching processes involved across 287 Delphi studies [46]. During the initial round, participants will be allowed to propose new, additional PREM items for consideration in subsequent Delphi rounds. To warrant inclusion in the final PREM, items must reach consensus across at least two Delphi rounds.

Suggested items will be included using the language of the participant unless editing is required for clarity. This will either be done through voting and feedback in subsequent rounds or checking directly with the participant as required. Consultation with the LEAG and peer researchers will be undertaken as necessary to guide decision making. All items will be reviewed for brevity and clarity in the cognitive debriefing process.

Following completion of the Delphi rounds, young trans Delphi panellists exclusively (not parent/carers or healthcare professionals who took part) will be invited to a post‐Delphi discussion group. Participants will be presented with a summary of the Delphi process, including items that were included and excluded. Final included PREM items will be reviewed and confirmed for inclusion and any excluded items still desired will be discussed by the group. Where appropriate, researchers will explain rating decisions underpinning these for deliberation to the group. The researchers will summarise the proceedings of this discussion group to the LEAG and any items that are contentiously included/excluded may be subject to change. This step represents the testing and refinement of Delphi insights through prioritising the insights of trans young people [35] whose lived experiences hold greater epistemic value within the context of this PREM. Because the LEAG are involved across the life of the project and will have a close understanding of all knowledge generated by the project, they will have the ability to reverse the exclusion of an item if they feel it is central to the findings from the scoping review and interview data. If this occurs, this will be made clear in the reporting of the Delphi study and a full justification will be provided.

## 8 Stage 4: Cognitive Debriefing Focus Groups

Two, 1.5 h cognitive debriefing [49] focus groups will be conducted with approximately seven trans young people aged 14 to 25 years living in Australia per group (*N* = 14). Trans young people who participate in Stage 2 interviews and indicate they are interested in participating in future related research will be invited, as will trans young people from the community. Cognitive debriefing is a method that can be used to assess clarity and comprehension, check relevance and appropriateness, identify ambiguity and misinterpretation, understand how participants rate a PREM to ensure it is in line with the intended constructs, and refine the item wording and format in order to maximise readability, sensitivity, and usability before piloting the PREM [50]. Cognitive debriefing is an important part of scale development and validation processes [51], and will generate valuable insights into the content validity of the measure (i.e., to what extent trans young people perceive the WOSAC PREM to be measuring the content and attributes that constitute WOSAC) and face validity (i.e., whether trans young people perceive the WOSAC PREM to be accurately measuring what they consider to be WOSAC).

Traditionally employed in a one‐on‐one, in‐person interview setting, contemporary researchers highlight that there is value in facilitating group‐based collective cognitive debriefing where a prospective measurement tool pertains to topics and experiences specific to minoritised identities, experiences, and backgrounds [50, 52]. Moreover, focus groups capture individualistic experiences cognitive processing of a given survey item based solely on one's own identities, experiences, backgrounds, and values, while also ensuring that items capture the collective realities of individuals with shared minoritised identities, backgrounds, and experiences [50]. A previous community‐based study has found that, compared to one‐to‐one interviews, cognitive debriefing focus groups reduce burden, thereby promoting more open‐ended dialogue among participants and yielding richer qualitative datasets [53]. Conducting these cognitive debriefing protocols in a focus group setting rather than individual interview environment honours the importance of collective learning and collective sense making that is a key feature of co‐production processes [35].

During cognitive debriefing focus groups, participants will be presented with information about WOSAC PREM item content validity and readability, and response options and scoring. Participants will be asked to reflect on what the items mean to them, including how they think about items, interpret items, and respond to items. The peer facilitator of these focus groups will interactively synthesise participants' accounts.

Candidate PREM items will be presented alongside several potential response scale options for trans young people to also express which they prefer, such as a 5‐point Likert scales where for example, ‘1 = Never and 5 = Always’. LEAG, CWG, and PSG input will also determine potential temporal reference ranges considered by the PREM (if applicable), for example a prompt beginning with ‘In the past 6 months…’, or ‘In the past 3 months…’ These options will be based on findings from preceding study stages with input from LEAG and PSG members.

Candidate PREM items will be modified to reflect focus group participants' suggestions regarding wording and preferred response options. LEAG members will be tasked with working together to develop an a priori hypothesis justifying how items should be ordered to capture WOSAC. These items will form the ‘refined candidate items’ comprising the PREM. The ordering of the items will make up the ‘candidate PREM measurement model’. Cognitive debriefing focus groups will discuss how the PREM will be administered (e.g., paper vs online format) and also ensure that final PREM items are not too difficult to read and comprehend. Discussion will clarify what each PREM item is ‘intended’ to capture. Focus group participants will also collaboratively discuss how scores for each PREM item ought to be interpreted.

## 9 Project Outcomes

By conducting Stages 1 to 4, the proposed project will co‐produce a validated PREM ready for use with trans young people attending healthcare services. This project will also produce support documents to aid interpretation, such as scoring and interpretation guidance.

## 10 Discussion

This protocol outlines a multi‐phase participatory research study which aims to co‐produce a PREM capturing trans young people's experiences of receiving primary healthcare and mental healthcare services. Through scoping review of qualitative accounts of marginalised adolescents' experiences of receiving healthcare, interviews with trans young people and healthcare professionals, and a modified e‐Delphi consensus survey with trans young people, their parents/carers, and their healthcare professionals, the newly generated PREM will enable monitoring and quality improvement of trans young peoples' experiences accessing healthcare services. Deliberate attention directed toward representing trans young people encountering intersecting systems of oppression will optimise the odds that the prospective PREM will capture elements of healthcare experiences pertinent to trans young people from multiple marginalised isocial positions.

The proposed PREM will be able to comprehensively evaluate the quality of the experiences of trans young people in healthcare settings, according to what trans young people value most. This will be invaluable because healthcare systems often find it difficult to meet the needs of trans young people [14], especially those who are multiply marginalised. There is an urgent need for healthcare services to affirm all aspects of trans young peoples lives, including but not limited to their gender [17, 18]. The real‐world use of the WOSAC PREM holds the potential to generate targeted insights and recommendations for service providers and healthcare settings to improve the quality of care provided to trans young people, including those overcoming interlocking systems of oppression such as trans young people of colour.

The prospective PREM will be used as part of a mixed methods evaluation of an integrated gender service model operating in primary youth mental healthcare services in Victoria, Australia. As part of this funded research study, the implementation of this PREM will be supplemented with an adjunct service‐facing implementation toolkit, service fidelity checklist, or similar, providing recommendations and reflection points for services to consider in striving toward promoting WOSAC in service provision to multiply marginalised trans young people. The PREM will also be trialled in paediatric gender services nationally throughout Australia as part of the Australian Research Consortium for Trans Youth and Children.

A key strength of this study will be its centring of the lived/living experience of multiply marginalised trans young people. The employment of trans peer researchers as well as the curation of a trans youth advisory group with cross‐cutting presence at weekly CWG meetings and bi‐monthly PSG meetings represents a strong governance structure which enables lived experience input into decision making throughout all project stages. This study will also benefit from the mixed use of quantitative, qualitative, and evidence synthesis methodologies and, moreover, a diverse and multi‐disciplinary team spanning peer researchers, participatory methodologists, mental health professionals, paediatricians, implementation scientists, and traditional researchers with broad clinical and research expertise in trans youth mental health.

Several limitations warrant caution when interpreting prospective study findings. This study will be conducted using the English language and this will automatically render trans young people who do not speak English as a first language in a systemically less opportune position to participate in the study. This limitation will also reduce the generalisability of the prospective WOSAC PREM to all trans young people from diverse multicultural and multifaith backgrounds. Though the LEAG and its centring of trans youth lived experience throughout this project represents a strength, it is also important to note that prospective LEAG members will likely be from relatively more privileged backgrounds because those trans young people with less social capital (e.g., awareness about participating in research, free time away from work and/or study, trans community connectedness) will be less likely to apply for a LEAG position. Trans young people who have had negative experiences of healthcare services and are thus more likely to hold multiple marginalised social positions may also be less likely to engage with this study.

This project will co‐produce and validate a PREM for evaluating the extent to which experiences of care affirm all aspects of the lives of trans young people in Australia, in ways including but not limited to their experiences of gender and transness. By enabling the identification of tangible healthcare experience factors which are not meeting the needs of trans young people, findings will improve the quality of affirming care provided to trans young people, particularly those who face intersecting barriers to care.

## Author Contributions

**Sasha Bailey:** methodology, writing – original draft, writing – review and editing, project administration, investigation. **Isabel Zbukvic:** conceptualisation, investigation, funding acquisition, methodology, writing – review and editing, supervision. **Alex Dalton:** investigation, methodology, writing – review and editing, project administration. **Steph Odoi:** investigation, methodology, writing – review and editing, project administration. **Emily Clarke:** writing – original draft, writing – review and editing, methodology, investigation. **K. A. McKercher:** conceptualisation, investigation, methodology, writing – review and editing, funding acquisition. **Ken Pang:** conceptualisation, investigation, funding acquisition, methodology, writing – review and editing. **Alessandra Chinsen:** methodology, writing – review and editing. **Maria Pallotta‐Chiarolli:** funding acquisition, conceptualisation, writing – review and editing, methodology. **Alan Bailey:** methodology, writing – review and editing. **Penelope Strauss:** funding acquisition, conceptualisation, writing – review and editing, methodology. **Kate Filia:** conceptualisation, funding acquisition, writing – review and editing, methodology. **Caroline Gao:** conceptualisation, funding acquisition, writing – review and editing, methodology. **Skye Barbic:** conceptualisation, methodology, writing – review and editing, funding acquisition. **Sarah Bendall:** conceptualisation, funding acquisition, writing – review and editing, methodology. **Sian Lloyd:** funding acquisition, methodology, writing – review and editing, conceptualisation. **Andrew Chanen:** conceptualisation, funding acquisition, writing – review and editing, methodology. **Nicholas Fava:** conceptualisation, funding acquisition, writing – review and editing. **Eóin Killackey:** conceptualisation, funding acquisition, writing – review and editing. **Susanne Prosser:** writing – review and editing. **Vikki Ryall:** writing – review and editing, methodology, conceptualisation, funding acquisition. **Lou Kerley:** writing – review and editing, methodology. **Corrinne T. Sullivan:** writing – review and editing. **Magenta Simmons:** conceptualisation, investigation, funding acquisition, writing – review and editing, methodology, project administration, supervision, resources.

## Ethics Statement

This study has received ethics approval from the University of Melbourne Human Research Ethics Committee (reference number: 2024‐29368‐61226‐7).

## Conflicts of Interest

Lou Kerley, Ken Pang, Magenta Simmons, and Isabel Zbukvic are members of the Australian Professional Association for Trans Health. Sasha Bailey, Lou Kerley, Ken Pang, Pip Buckingham and Magenta Simmons are researchers in the Australian Research Consortium for Trans Youth and Children (ARCTYC). Ken Pang is also a member of the World Professional Association for Trans Health, an associate editor of the journal Transgender Health, and a member of the NHMRC Gender Guidelines Development Committee.

## Data Availability

Data sharing not applicable to this article as no datasets were generated or analysed during the current study.
